# Mimicry and automatic imitation are not correlated

**DOI:** 10.1371/journal.pone.0183784

**Published:** 2017-09-06

**Authors:** Oliver Genschow, Sofie van Den Bossche, Emiel Cracco, Lara Bardi, Davide Rigoni, Marcel Brass

**Affiliations:** 1 Social Cognition Center Cologne, University of Cologne, Cologne, Germany; 2 Department of Data Analysis, Ghent University, Ghent, Belgium; 3 Department of Experimental Psychology, Ghent University, Ghent, Belgium; University of California Los Angeles, UNITED STATES

## Abstract

It is widely known that individuals have a tendency to imitate each other. However, different psychological disciplines assess imitation in different manners. While social psychologists assess mimicry by means of action observation, cognitive psychologists assess automatic imitation with reaction time based measures on a trial-by-trial basis. Although these methods differ in crucial methodological aspects, both phenomena are assumed to rely on similar underlying mechanisms. This raises the fundamental question whether mimicry and automatic imitation are actually correlated. In the present research we assessed both phenomena and did not find a meaningful correlation. Moreover, personality traits such as empathy, autism traits, and traits related to self- versus other-focus did not correlate with mimicry or automatic imitation either. Theoretical implications are discussed.

## Introduction

Individuals imitate a wide range of different behaviors including facial expressions [1], characteristics of language [2–5], emotions [1,6], postures [7], gestures [8], complex action patterns [9] or simple movements [10–14]—to name just a few examples. Research from the last decade suggests that imitative behavior may function as a “social glue” (e.g., [15,16]) in the sense that it strengthens human bonds by increasing pro-social behavior [17], feelings of affiliation [18], or liking for each other [6].

Within the domain of imitation, social psychologists are applying different paradigms than cognitive psychologists. With reference to the chameleon effect, social psychologists investigate mimicry—individuals’ tendency to imitate others in social settings. Mimicry has most often been investigated by applying naturalistic paradigms that measure the frequency of imitation in interactions between a participant and a confederate (e.g., [6,16,19–22]). For example, Chartrand and Bargh [6] let participants engage in a card-sorting task with a confederate who repeatedly touched his face in one half of the experiment and repeatedly waggled his food in the other half of the experiment. Importantly, participants’ behavior was videotaped during the experiment so that the researchers could code how often participants engaged themselves in face touching and food waggling. The typical result of such a paradigm is that participants more often touch their face than waggle their foot when the confederate touches his face and vice versa when the confederate waggles his foot. Besides such one-to-one interactions, researchers have also used adapted versions (e.g., [11–13,18,21,22,23,24–27]) in which participants observe a video of a model who engages in two classes of different behaviors (e.g., nose touching vs. hair touching). Similar to the aforementioned studies, participants are videotaped while they watch the videos. Afterwards, coders measure the degree of mimicry by coding how often participants performed the two target actions themselves.

In contrast to such naturalistic paradigms, researchers in cognitive psychology focus on automatic imitation, which can be seen as the laboratory model of imitation [28]. In a typical experiment, participants respond over many trials with two different movements to two different symbolic cues while seeing a congruent movement, an incongruent movement, or no movement on a computer screen. For example, in the task developed by Brass, Bekkering, and Prinz [10], participants have to lift their index or middle finger in response to the number ‘1’ or ‘2’. At the same time, participants also see a hand performing either the same finger movement (i.e. congruent), the other finger movement (i.e. incongruent), or no finger movement (i.e. neutral). Automatic imitation in this paradigm refers to the finding that individuals respond faster and with fewer errors to congruent trials than to incongruent trials (i.e., *congruency effect*). However, besides the congruency effect, researchers can also compute the *facilitation effect—*individuals’ tendency to respond faster and with fewer errors to congruent trials than to neutral trials. Finally, the experimental setup allows measuring individuals’ tendency to inhibit imitative responses (i.e., *interference effect*). That is, individuals respond slower and with more errors to incongruent trials than to neutral trials (e.g., [10,29]).

In the literature, the terms mimicry and automatic imitation are often used synonymously [28]. Thus, there seems to be wide agreement that mimicry and automatic imitation are correlated. But is this indeed the case? The answer to this fundamental question may depend on whether one compares the methods or the theoretical assumptions of mimicry and automatic imitation. While a methodological analysis suggests that the two paradigms measure different constructs, a theoretical analysis instead points towards a common ground.

### Methodological differences between mimicry and automatic imitation

Paradigms on mimicry and automatic imitation differ in many methodological aspects (for an overview see Table 1). First, mimicry tasks have higher ecological validity than automatic imitation tasks, because mimicry, but not automatic imitation, assesses imitation in natural settings (cf. [28]). Second, the two tasks differ in terms of dependent variables. That is, paradigms on automatic imitation measure responses in the form of reaction times and error rates to congruent and incongruent observed movements on a trial-by-trial basis. In mimicry paradigms, however, researchers count how often participants engage in a certain behavior within a predefined time period and then calculate a sum score that indicates how often the confederate was imitated. Third, the two imitation paradigms differ in the degree to which participants become aware about the effect. Mimicry effects remain unaware for participants (cf. [30]). That is, in posttest interviews, participants usually report no awareness of the model’s focal behavior (e.g., nose touching), no awareness of an intention to mimic, and no awareness that they mimicked the confederate’s behavior (e.g., [6]). In contrast, in automatic imitation paradigms more conscious awareness seems to be involved. Although it has not yet been empirically tested, it is our experience that participants are well aware that the other person’s finger movements influence their performance. Fourth, the two paradigms differ in the psychological processes needed to work on the task. While automatic imitation tasks need explicit cognitive control and executive functioning (e.g., [31]), mimicry tasks need less explicit cognitive control because mimicry is unconsciously driven and no actions need to be suppressed [6].

**Table 1:** Differences between mimicry and automatic imitation tasks.

Task characteristics	Mimicry	Automatic imitation
Ecological validity	high	low
Dependent variable	Subjective ratings of executed actions	Reaction times and error rates
Awareness	low	high
Cognitive control	low	high

### Theoretical similarities between mimicry and automatic imitation

Although the paradigms used in research on mimicry and automatic imitation differ in several aspects, there are also important similarities with regard to the theoretical assumptions and moderators of mimicry and automatic imitation.

#### Theoretical assumptions

Irrespective of how imitation is assessed, it is generally agreed that automatic imitation and mimicry are both based on shared representations of observed and executed actions. For example, ideomotor theory [32–35]—a theory shaped in cognitive psychology—proposes that the visual image of an action is part of its motor representation. As a result, the observation of a certain action primes the execution of the same action. A similar proposition can be derived from research on mimicry, which puts forward the so-called perception–behavior link [6,36–38]. This link implies that the observation of an action evokes the same representation as the execution of that action. This common representation then increases the likelihood of the execution of the perceived action. Regardless of its framing, the idea that the observation of an action leads to the activation of the corresponding motor plan in the observer has now been confirmed extensively in behavioral studies (e.g., [10,29,39,40]), fMRI studies (e.g., [41,42]), motor TMS studies (e.g., [43,44]), and single-cell recordings in both monkeys [45] as well as humans [46].

#### Moderating influences

Past research on moderating influences suggests that similar factors facilitate mimicry and automatic imitation. For example, research has repeatedly found that empathy positively correlates with different kinds of imitation paradigms [47–49]. Other research suggests that the link between empathy and imitation is specific to a subfactor of empathy—namely perspective taking. In this respect, it has been shown that watching actions from the first-person perspective increases imitation as compared to the third-person perspective [12,50,51]. Similarly, when assessing perspective taking as a personality trait with the Interpersonal Reactivity Index (IRI; [52]), research found that the ability to take another person’s perspective increases mimicry [6,53] as well as automatic imitation [54, 55].

Another often reported facilitator of imitation is the degree to which individuals focus on others as compared to themselves. To explore the role of self-construal [56], it has been tested whether individuals who perceive themselves as dependent on others (i.e., interdependent self-construal) imitate others more strongly than individuals who perceive themselves as unique individuals (i.e., independent self-construal). For example, van Baaren, Maddux, Chartrand, De Bouter, and van Knippenberg [57] found that priming an interdependent, as compared to an independent self-construal, increases mimicry. Moreover, the authors demonstrated that individuals from eastern societies, who are known for their interdependent self-construal, mimic others more strongly than individuals from western societies, who are known for their independent self-construal. Similar findings have been found in research on automatic imitation. Hogeveen and Obhi [58], for instance, used the same priming as van Baaren and colleagues and found larger congruency effects following an interdependent self-construal manipulation, as compared to an independent self-construal manipulation. Relatedly, research has found reduced automatic imitation effects when increasing the self-focus of participants by letting them sit in front of a mirror [59].

Another moderator that has been investigated is autism. For example, Cook, Swapp, Pan, Bianchi-Berthouze and Blakemore [60] found reduced automatic imitation effects in individuals with autistic spectrum disorder as compared to healthy controls. Similarly, research has repeatedly found decreased intentional mimicry for individuals with high autistic traits (for a review, see [61]).

Based on the above-reviewed literature, it has been claimed that certain personality factors influence imitation. That is, high empathic persons and high perspective-takers, individuals with an interdependent self-construal, as well as people scoring low on autistic traits should imitate others more strongly. However, the empirical evidence for this claim is less clear than what has often been assumed. First, research on autism did not only find reduced automatic imitation for individuals with high autistic traits, but also intact automatic imitation [62–64] or even increased automatic imitation [65]. Second, some researchers were not able to replicate the findings on the moderating role of empathy on imitation [54,66]. Third, although it is claimed that self-construal moderates imitation, there is to the best of our knowledge no study that actually showed self-construal as part of a personality trait to moderate imitation.

### Present research

Taken together, the literature on imitation does not offer a clear answer to the question whether mimicry and automatic imitation are correlated and to which degree personality traits moderate imitation. Theoretical models on the mechanisms of imitation suggest that mimicry and automatic imitation are linked to each other and that certain personality traits should moderate mimicry and automatic imitation in the same way. However, some studies did not find support for these moderating influences. Moreover, methodological differences between mimicry and automatic imitation suggest that the two tasks might not be correlated. Therefore, in the present research we tested for the first time whether mimicry and automatic imitation are correlated and to which degree personality traits that have previously been linked to mimicry and automatic imitation moderate both phenomena. To this end, we ran a highly powered study with two hundred participants. To assess mimicry and automatic imitation, we applied the most often reported tasks in the literature.

## Method

### Ethics statement

The study was conducted in accordance with the ethical standards of the 1964 Declaration of Helsinki and approved by the rules of the Institutional Review Board from the Faculty of Psychology and Educational Science of Ghent University. All participants provided informed consent at the beginning of the experiment and were informed that participation was voluntary and that all answers were processed and stored anonymously.

#### Data availability statement

The data file of the study is available from the Open Science Framework database. The URL necessary to access our data is: https://osf.io/v3afy/.

### Participants

In return for partial course credit, two hundred students from Ghent University (Belgium) participated in this study. All participants reported normal or corrected-to-normal vision and hearing as well as confirmed to speak and comprehend Dutch. Prior to data analysis, we excluded two participants. One participant did not allow us to analyze her recorded videotape and was thus excluded from all analyses. Another participant was excluded, because she misunderstood the task. Moreover, two participants who had a mimicry score or a congruency effect higher than 4 SD above the sample mean were identified as outliers and discharged as well (note: leaving these outliers in the sample does not change the results). The reason why we discharged these outliers is that the removal of outliers leads to more accurate correlations between measures—that is they become closer to the population correlation (e.g., [67]). Thus, the final sample contained 196 participants (135 women, 61 men) with an age ranging from 17 to 44 (*M* = 18.74; *SD* = 2.74).

### Procedure

After being welcomed, participants were seated in separate cubicles. The setup of each cubicle consisted of an Asus Eee PC 1215N laptop containing an integrated webcam, an external 17-inch Dell monitor, two speakers and an apple MB110FN/B AZERTY mac keyboard. Participants sat at a viewing distance of approximately 35 cm to the monitor. After participants were seated, they signed a written informed consent, and then ran through the mimicry and automatic imitation task. The order of these two tasks was counterbalanced between participants. Afterwards, participants filled in a couple of questionnaires measuring personality traits and indicated demographic data. At the end, the experimenter fully debriefed participants. Thereby, she told participants that they were videotaped during the experiment and asked whether we were allowed to use the videos for scientific purposes. Finally, participants were thanked and dismissed.

### Materials/ Stimuli

#### Mimicry task

**Fig 1:**
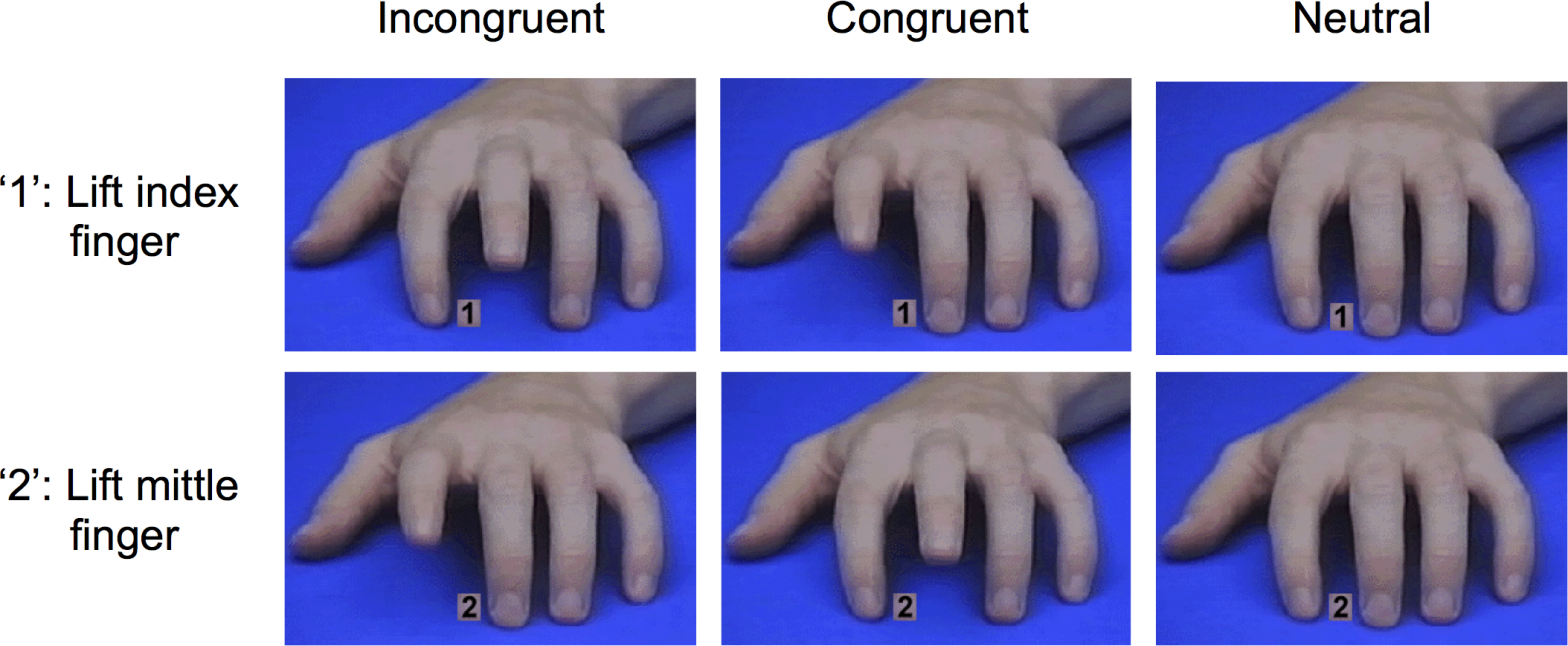
Overview of incongruent, congruent and neutral trials in the automatic imitation task.

The mimicry task was an adaption of previously used video-based mimicry tasks [e.g., 11-13,18,21,22,23,24-27]. Participants were told that they were going to watch two video clips of a model reading a story and that they should listen carefully to the story as questions about the text had to be answered at the end of the experiment. Participants then watched two video clips in which a female model read a story about a rabbit from the Dutch children book titled “365 konijnenverhaaltjes voor het slapengaan (365 rabbit stories to go off to sleep)” [68]. Each video lasted 10 minutes. In both videos, the model engaged in a specific action every 20 seconds. That is, in one video she was touching her nose and in the other video she was touching her hair (see Fig 1 for screenshots of the videos). The sequence of nose touching versus hair touching videos was counterbalanced between participants. In line with the cover story, participants had to answer ten story-related questions about the story after watching the videos. Throughout the task, a webcam was used to videotape participants. In order to prepare data for analyses, a coder blind to the conditions counted for each video how often participants engaged in nose touching and hair stroking actions. To cross-validate the coding, a second coder coded the videos of 50 randomly selected participants. The intra-class correlation coefficient was *r* = .77, *p* < .001.

#### Automatic imitation task

The automatic imitation task was based on Brass and colleagues’ [10] task and was programmed using Tscope5 software [69]. In the beginning of each trial, participants were instructed to press down the ‘G’ and ‘H’ key with their right index and middle finger. Then, a fixation cross appeared for 500 ms. Afterwards, a picture of a mirrored left hand in resting position was presented for 200 ms. This picture was followed by a second picture that was presented for a maximum of 2000 ms or until participants gave a response. Depending on the condition, the picture either indicated an index finger movement, a middle finger movement, or no movement. At the same time, a number was displayed between the model’s middle and index finger. Participants were instructed to lift their index finger as fast as possible in response to the number ‘1’ and to lift their middle finger as fast as possible in response to the number ‘2’ (see Fig 1 for an overview of the stimuli). This setup resulted in three different trials: In congruent trials participants responded with the same finger as the model, in incongruent trials participants responded with another finger than the model, and in neutral trials the participants responded with a finger while the model did not respond at all.

*T*he automatic imitation task contained a practice and an experimental phase. The practice phase contained 12 trials. The experimental phase contained 360 trials resulting in 120 congruent trials, 120 incongruent trials and 120 neutral trials. In the experimental phase, participants were allowed to take a break after every 90 trials. In order to reduce the influence of spatial compatibility, participants were instructed to place their right hand rotated 90° counterclockwise to the model’s hand displayed on the screen (e.g., [70,71,72]).

To prepare the data for analysis, we removed extremely slow reaction times. That is, latencies below (1.20%) and above (0.05%) 3 SD of the participants’ mean.

#### Personality scales

In order to test the degree to which personality traits that have previously been reported to moderate imitation correlate with mimicry and automatic imitation, we administered a couple of questionnaires.

**Perspective taking and empathy:** To assess perspective taking and empathy, we administered the Dutch version [73] of the Interpersonal Reactivity Index (IRI; [52]). The IRI contains 28 items that were scored on 5-point rating scales ranging from 1 (*does not describe me well*) to 5 (*describes me very well*). The IRI is divided into four dimensions, each containing 7 items: Besides Perspective Taking (Cronbach’s α = .68), it administers Fantasy (Cronbach’s α = .86), Empathic Concern (Cronbach’s α = .64), and Personal Distress (Cronbach’s α = .79). In order to prepare data for analyses, we computed mean scores of all subscales as well as a mean score over all subscales in order to compute a total score of empathy (Cronbach’s α = .83).

**Self- versus other focus:** We assessed multiple scales to get insight into participants’ focus on themselves versus others. First, we used a self-translated Dutch version of the Self-Construal Scale (SCS; [74]). The SCS measures the strength of an individual’s interdependent and independent self-construal. These two self-construals are conceptualized as reflecting the emphasis on connectedness and relations often found in non-Western cultures (interdependent) and the separateness and uniqueness of the individual (independent) stressed in Western societies. The SCS contains 30 items, divided into an independence self-construal (Cronbach’s α = .70) and an interdependence self-construal (Cronbach’s α = .72). Participants answered on a 7-point scale ranging from 1 (“*strongly disagree*”) to 7 (“*strongly agree*”) to which degree they agreed to statements related to their self. In order to compute a total score of self-construal, we subtracted the interdependence self-construal subscale from the independence self-construal subscale. High values indicate a strong relative independence self-construal.

Second, we administered the Dutch version of the short Individualism and Collectivism Scale (ICS; [75]). The shortened version of the ICS contains eight items, divided into four subscales: Horizontal Individualism, Horizontal Collectivism, Vertical Individualism and Vertical Collectivism. For individuals high on horizontal collectivism, the well-being of their in-groups (e.g., family, tribe, coworkers, nation) is of central importance. However, they do not feel subordinate to their in-groups. In contrast, individuals high on vertical collectivism submit to the norms of their in-groups and are even willing to sacrifice their personal identities for their in-groups. Horizontal individualists, however, are characterized by seeking individuality rather than distinctiveness by doing their own thing and not to compare themselves with others. Vertical individualists are especially concerned with comparing themselves with others. That is, they believe that competition is the law of nature, and they desire to win in all kinds of competitions. Participants indicated on 7- point scales ranging from 1 (“*definitely not true*”) to 7 (“*definitely true*”) the degree to which the 8 statements apply to them. To compute a total score of the scale, we subtracted the mean of the collectivism items (Cronbach’s α = .45) from the mean of the individualism items (Cronbach’s α = .63). A high score reflects a relative high individualism as compared to collectivism.

Third, we assessed participants’ need to belong. Need to belong refers to people’s desire for interpersonal acceptance and belonging and is associated with greater sensitivity to interpersonal cues in order to foster connections with others [76]. In order to assess need to belong, we applied a self translated Dutch version of the Need to Belong Scale (NTBS; [77]). Participants indicated the degree to which each of the 10 statements applied to them on a 5-point scale (1 = *strongly disagree*, 5 = *strongly agree*). Example items of the scale are “I try hard not to do things that will make other people avoid or reject me,” and “I want other people to accept me.” Cronbach’s alpha for the NTBS was α = .85.

*Autism-Spectrum Quotient*. To assess a participant’s autistic-like traits, we used the Dutch version [78] of the Autism-Spectrum Quotient (AQ; [79]). The AQ has been found to be strongly predictive of who receives a diagnosis of autism spectrum disorder in a clinic setting [80]. The AQ contains 50 items, divided into five subscales: social skill (Cronbach’s α = .66), attention switching (Cronbach’s α = .56), communication (Cronbach’s α = .32), imagination (Cronbach’s α = .63) and attention to detail (Cronbach’s α = .72). Participants answered on 5-point scales (1 = *strongly disagree*; 5 = *strongly agree*) to which degree statements about themselves apply to them. The subscale scores were averaged into a single total score (Cronbach’s α = .71).

#### Demographic data and further personality scales

Besides gender and age, we assessed further scales that had not yet been tested to moderate mimicry and automatic imitation. That is, we assessed participants’ engagement in social activities, socioeconomic status, number of friends, learning styles and regulatory focus.

*Social engagement*. To assess social engagement, participants answered with *yes* or *no* whether they had been engaging in the following social activities: “donating blood,” “registered as stem cell donor,” “engagement in voluntary work,” “member of a charitable institution (e.g., UNICEF, Greenpeace, etc.),” and “donating for a charitable institution (e.g., UNICEF, Greenpeace, etc.).” In order to compute a total score we summed up the amount of yes-answers participants gave on these items.

*Socioeconomic status*. We assessed participants’ objective as well as subjective socioeconomic status (SES; [81]). To assess the subjective SES, participants responded to the following six statements on a 5-point scale (1 = *strongly disagree*; 5 = *strongly agree*): “My family usually had enough money for things when I was growing up,” “I grew up in a relatively wealthy neighborhood,” “I felt relatively wealthy compared to the other kids in my school,” “I have enough money to buy things I want,” “I don’t worry too much about paying my bills,” “I don’t think I’ll have to worry about money too much in the future.” Cronbach’s alpha for the mean score of this subjective SES was α = .77.

To assess participants’ objective SES, they indicated on two 6-point scales (1= *€1*,*000 or less*; 2 = *€1*,*001 - €2*,*000*; 3 = *€2001 - €3*,*000*; 4 = *€3*,*001 - €4*,*000*; 5 = *€4*,*001 - €5*,*000*, 6 = *more than €5*,*000*) their income per month: “What was your annual gross household income when you were growing up? ,” “What is your present annual gross household income?” In addition, participants indicated on three 10-point scales the following questions: “What is the highest educational level completed by your father?” “What is the highest educational level completed by your mother?” “What is your highest educational level?”. To compute a total objective SES score, we calculated the mean of all items. Cronbach’s alpha of the objective SES was *α* = .62.

**Amount of friends:** To have some further indication about participants’ social life, they were asked how many friends they have. In addition, they also gave an estimation of their number of Facebook friends, in case they had Facebook.

**Learning style:** With a self-translated Dutch version, we assessed participants’ learning style [82]. The scale contained eight items, divided into four subscales: Active Learning, Concrete Experience, Reflective Observation, Abstract Conceptualization and Active Experimentation. All items were answered on a 5-point scale (1 = *does not describe me well*; 2 *= describes me very well*).

**Regulatory Focus:** We also assessed participants’ regulatory focus. Regulatory Focus Theory [83] distinguishes between the promotion focus, which regulates nurturance needs and goals related to aspiration and accomplishment (i.e., ideals), and the prevention focus, which regulates security needs and goals related to safety and responsibilities (i.e., oughts). We assessed the Dutch version (cf. [84]) of the 18-item regulatory focus scale developed by Lockwood et al. [85]. Participants indicated on 7-point scales (1 = *definitively not true*; 7 = *definitively true*) how different promotion- and prevention-related statements apply to them. In line with previous research [85–88], we computed a difference score by subtracting the score of the prevention focus subscale (Cronbach’s α = .70) from the promotion focus subscale (Cronbach’s α = .67). High values indicate a relative strong promotion focus compared to a prevention focus.

## Results

To test our hypotheses we conducted inference statistics using SPSS. In addition, we applied Bayesian statistics using JASP, an open source statistical package [89]. Specifically, we report the Bayes factors (BF) calculated with the default JASP priors. BF_10_ gives the ratio with which the alternative hypothesis is favored over the null hypothesis (i.e., larger BFs argue in favor of the alternative hypothesis; see [90] for an overview), whereas BF_01_ gives the ratio with which the null hypothesis is favored over the alternative hypothesis (i.e., a larger BFs argue in favor of the null hypothesis; see [90] for an overview). BF_01_ is defined as the inverse of BF_10_.

### Mimicry

#### Mimicry

**Fig 2:**
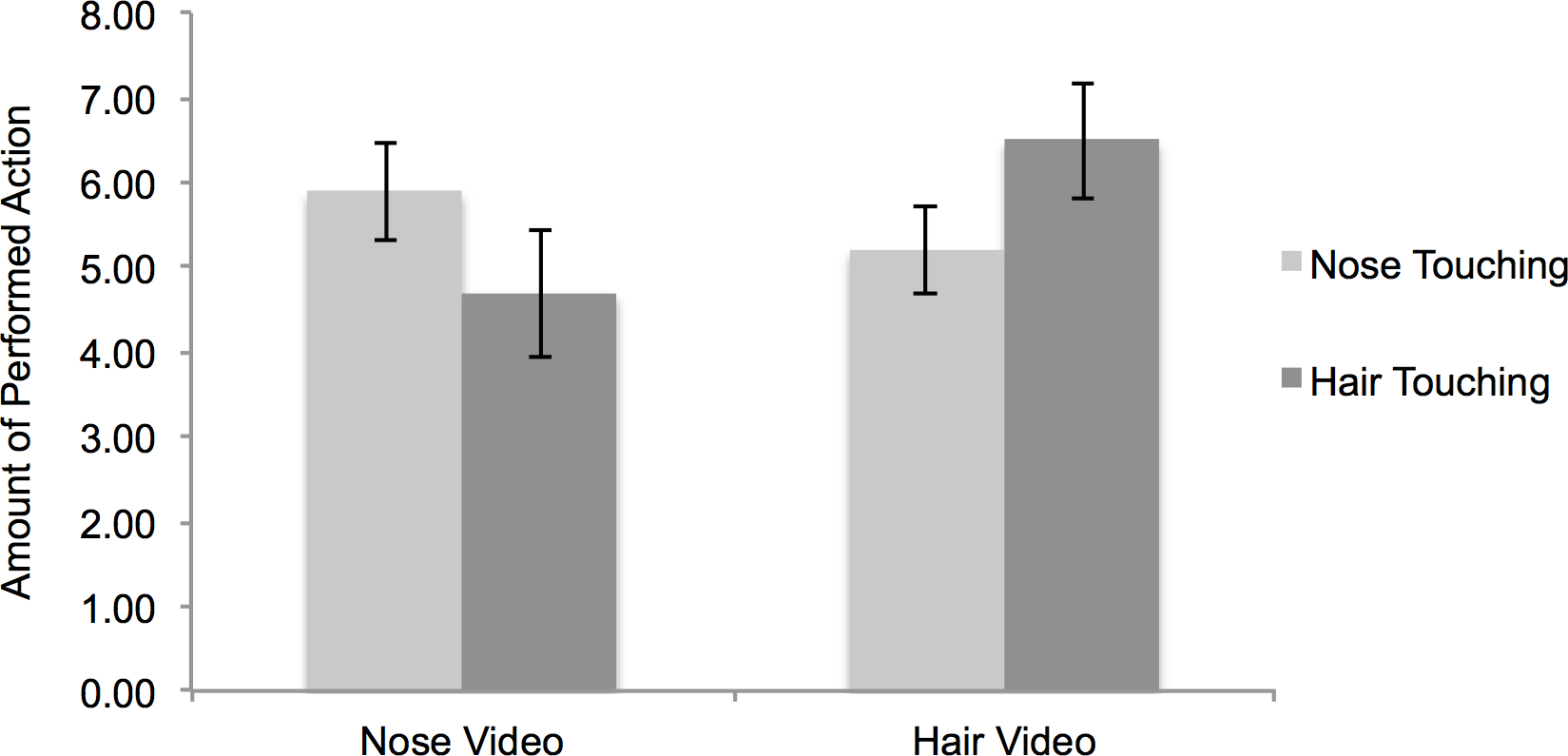
Amount of performed actions in the mimicry task. Error bars represent standard errors of the mean.

First, we ran a 2 (observed video: nose touching video vs. hair touching video) x 2 (performed action: nose touching action vs. hair touching action) repeated measures ANOVA to test the presence of a mimicry effect. The ANOVA yielded no significant main effects, *F*(1,195) < 1.54, *p* > .21. However, more importantly, we found a significant interaction between observed video and performed action, *F*(1,195) = 11.69, *p* = .001, *η*^*2*^_*p*_= .06 indicating the presence of a substantial mimicry effect (see Fig 2 for means).

In an additional analysis, we calculated a nose-mimicry score by subtracting the amount of nose touching actions in the hair video from the amount of nose touching actions in the nose video. Likewise, we computed a hair-mimicry score by subtracting the amount of hair touching actions in the nose video from the amount of hair touching actions in the hair video. In order to compute an overall mimicry score, we averaged the nose-mimicry score and the hair-mimicry score. To test the strength of this mimicry effect, we then conducted a Bayesian *t*-test in which we tested the hypothesis that the mimicry score (*M* =1.24; *SD* = 5.09) was larger than 0. The Bayesian *t*-test yielded BF_10_ = 43.12, indicating that the data is 43.12 times more likely to have occurred under the alternative hypothesis than under the null hypothesis. A Bayes factor < 100 and > 30 is conventionally considered to be very strong evidence [91].

#### Reliability

In a second analysis we tested the split-half reliability of the mimicry effect. That is, we first computed two different mimicry scores. One score was calculated from the even minutes and the other score was calculated from the odd minutes. In order to test the reliability of the mimicry task, we computed the Spearman-Brown coefficient. The coefficient was negative, ρ* = -.11 suggesting non-reliability.

In an additional analysis we computed a mimicry score for hair actions and a mimicry score for nose actions. The mimicry score for hair actions was computed by subtracting participants’ hair actions during the nose video from participants’ hair actions in the hair video. Likewise, the mimicry score for nose actions was computed by subtracting participants’ nose actions in the hair video from participants’ nose actions in the nose video. The Spearman-Brown coefficient for these two mimicry scores was negative, ρ* = - .59, suggesting non-reliability. This indicates that the low reliability is not restricted to one of the two actions. Moreover, we computed separately for even and odd minutes a nose-mimicry score and a hair-mimicry score. Spearman-Brown coefficients were ρ* =.13 for nose-mimicry and ρ* =.15 for hair-mimicry indicating low reliability.

### Automatic imitation

#### Latencies

**Fig 3:**
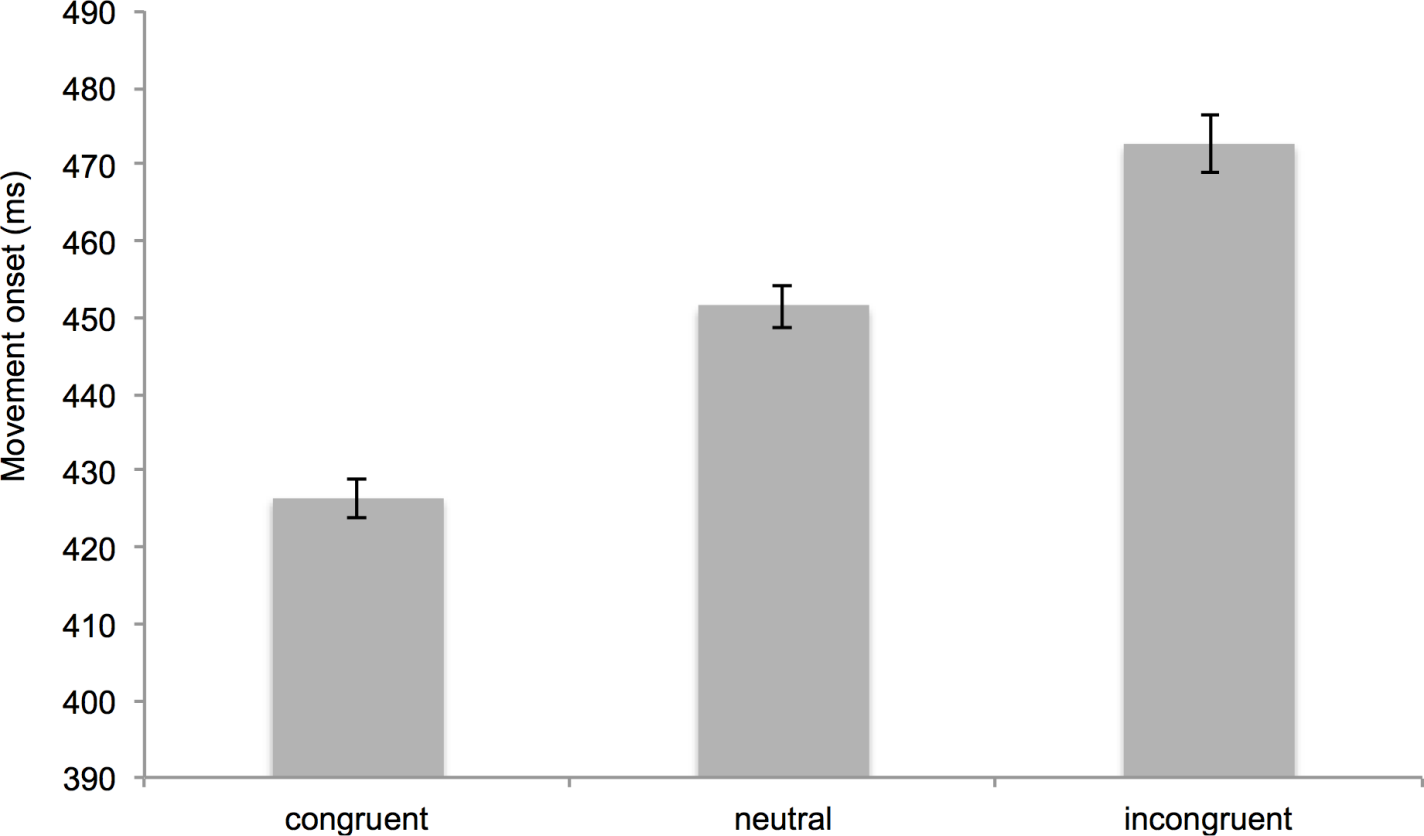
Mean reaction times of the automatic imitation task separated by condition. Error bars represent standard errors of the mean.

Fig 3 illustrates the results for the latencies. With respect to the *congruency effect*, the results indicated that individuals responded faster in congruent trials (*M* = 426.46, *SD* = 36.78) than in incongruent trials (*M* = 472.64, *SD* = 51.78), *t*(195) = 21.83, *p* < .001, *d* = 1.56. Similarly, the *facilitation effect* was significant. That is, participants responded faster in congruent trials (*M* = 426.46, *SD* = 36.78) than in neutral trials (*M* = 451.45, *SD* = 40.52), *t*(195) = 22.17, *p* < .001, *d* = 1.58. Moreover, the *interference effect* was significant suggesting that participants responded faster in neutral trials (*M* = 451.45, *SD* = 40.52) than in incongruent trials (*M* = 472.64, *SD* = 51.78), *t*(195) = 15.05, *p* < .001, *d* = 1.08.

In additional analyses, we tested the strength of the automatic imitation effects with Bayesian *t*-tests. Specifically, we conducted Bayesian *t*-tests and tested the hypothesis that the congruency effect (*M* = 46.19, *SD* = 29.62), the facilitation effect (*M* = 25.00, *SD* = 15.78), and the interference effect (*M* = 21.19, *SD* = 19.71) were larger than 0. The Bayesian *t*-tests yielded BF_10_ = 1.18·10^51^ for the congruency effect, BF_10_ = 1.01·10^52^ for the facilitation effect and BF_10_ = 3.22·10^31^ for the interference effect. A Bayes factor > 100 is conventionally considered to be extreme evidence [91].

#### Error rates

**Fig 4:**
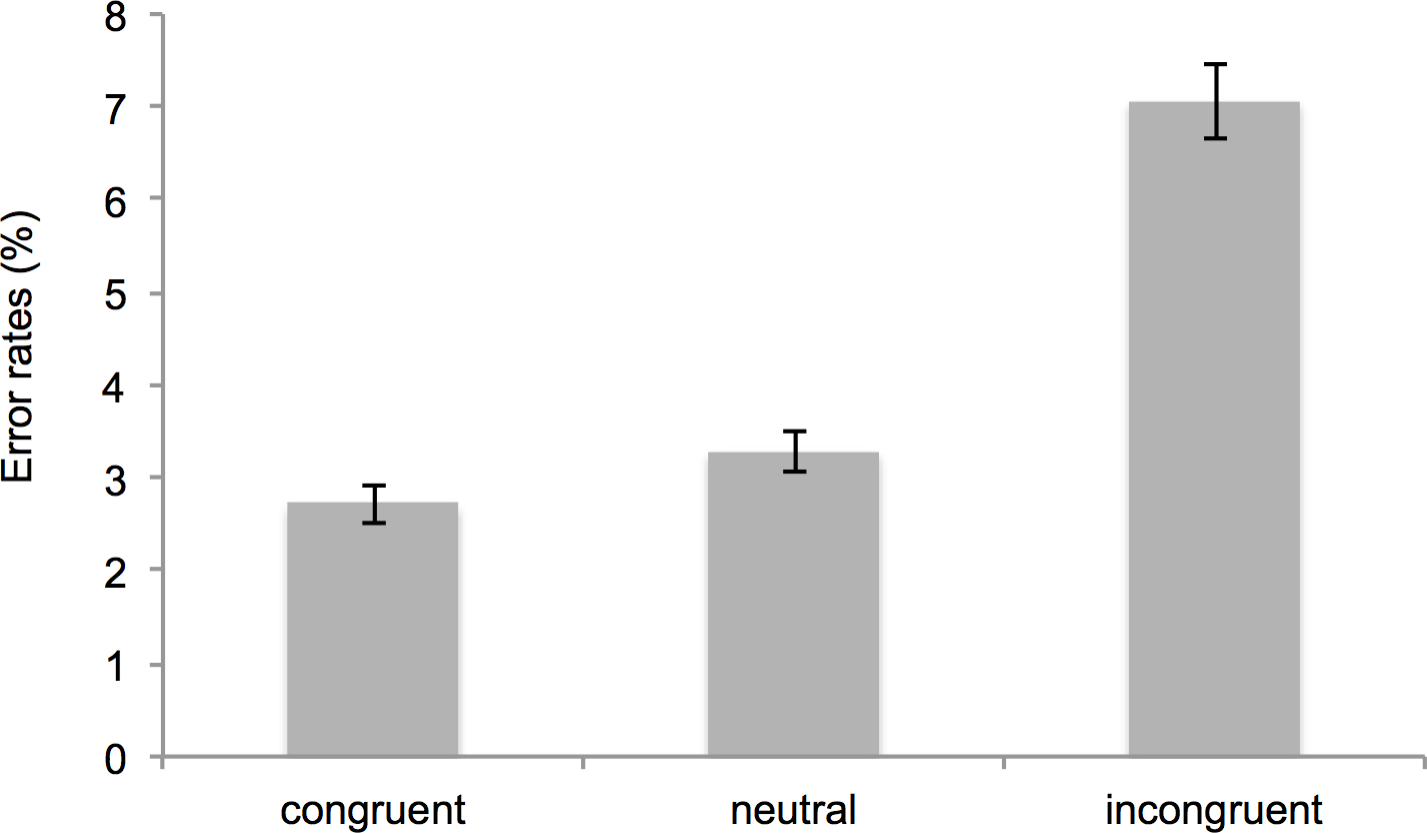
Mean error rates within the automatic imitation task separated by condition. Error bars represent standard errors of the mean.

As can be seen in Fig 4, the results for the error rates were in line with the results for the latencies. That is, participants made fewer errors in congruent trials (*M* = 2.72%, *SD* = 3.12), than in neutral trials (*M* = 3.29%, *SD* = 3.01), *t*(195) = 3.69, *p* < .001, *dz* = 0.26, and in incongruent trials, (*M* = 7.07%, *SD* = 5.72), *t*(195) = 13.74, *p* < .001, *d* > 0.98. Moreover, participants committed fewer errors in neutral trials (*M* = 3.29%, *SD* = 3.01) than in incongruent trials (*M* = 7.07%, *SD* = 5.72), *t*(195) = 11.64, *p* < .001, *d* = 0.83.

Bayesian *t*-tests testing the hypothesis that the congruency effect (*M* = 4.34 *SD* = 4.43), the facilitation effect (*M* = 0.57 *SD* = 2.17), and the interference effect (*M* =3.77 *SD* = 4.54) were larger than zero yielded BF_10_ = 3.68·10^27^ for the congruency effect, BF_10_ = 105.05 for the facilitation effect and BF_10_ = 1.94·10^21^ for the interference effect. A Bayes factor > 100 is considered to be extreme evidence [91].

#### Reliability

In a further analysis, we tested the split-half reliability of the automatic imitation task. First, we computed the congruency effect, the facilitation effect, and the interference effect for even trials and for odd trials separately for reactions times and for error rates. Second, we computed the Spearman-Brown coefficient in order to test the reliability of the automatic imitation task. For reaction times, the results yielded ρ* = .86 for the congruency effect, ρ* = .50 for the facilitation effect, and ρ* = .68 for the interference effect. For error rates, the results yielded ρ* = .61 for the congruency effect, ρ* = .001 for the facilitation effect, and ρ* = .62, for the interference effect.

### Relationship between mimicry and automatic imitation

To test the relationship between mimicry and automatic imitation, we ran correlational analyses and will report both the corresponding *p*-values and BFs. In order to test the relationship between mimicry and automatic imitation, we correlated the mimicry score with all the scores of the automatic imitation task (i.e., congruency effect, facilitation effect, and interference effect for reaction times as well as for error rates). However, none of the correlations involving the mimicry score reached significance, *r* < - .13 *p* > .08 (for an overview of all correlations, see Table 2). We then computed the BFs of all correlations to investigate whether the null hypothesis (i.e., mimicry and automatic imitation are not positively correlated) could be supported. When correlating the mimicry score with the reaction time scores of the automatic imitation task, the analyses yielded BF_01_ = 29.59 for the congruency effect, BF_01_ = 25.06 for the facilitation effect, and BF_01_ = 27.15 for the interference effect. When correlating the mimicry score with the error rates of the automatic imitation task, the analyses yielded BF_01_ = 9.51 for the congruency effect, BF_01_ = 9.08 for the facilitation effect, and BF_01_ = 10.55 for the interference effect. These results indicate moderate to strong evidence for the null hypothesis, which confirms that mimicry and automatic imitation are not positively correlated (cf., [91]).

**Table 2:** Intercorrelations between the mimicry score and all different automatic imitation scores.

	1.	2.	3.	4.	5.	6.	7.
1. Mimicry	—	-0.123	-0.096	-0.108	0.014	0.018	0.005
2. Congruency RT		—	0.789 ***	0.871 ***	0.322 ***	-0.019	0.323 ***
3. Facilitation RT			—	0.385 ***	0.346***	-0.053	0.363 ***
4. Interference RT				—	0.207**	0.014	0. 195 **
5. Congruency ER					—	0.192 **	0.883 ***
6. Facilitation ER						—	-0.290 ***
7. Interference ER							—

* *p* < .05

** *p* < .01

*** *p* < .001

*Note*. RT = Reaction Time; ER = Error Rate

### Relationship between personality traits and mimicry as well as automatic imitation

In further correlation analyses, we tested whether mimicry and automatic imitation correlated with the assessed personality traits and demographic data. That is, we ran three separate correlational analyses. In the first analysis, we ran a correlation analysis between the mimicry score and all assessed personality scales as well as demographic data. In the second analysis, we correlated all reaction-time-based scores from the automatic imitation task (i.e. congruency effect, facilitation effect, and interference effect) with the assessed personality scales and demographic data. Finally, in the third analysis, we calculated the same correlations for the error-rate-based scores of the automatic imitation task. For each of the three correlational analyses, we corrected for multiple comparisons by the means of FDR [92]. An overview of all the correlations is provided in Table 3.

#### Relation between mimicry and personality scales as well as demographics

The correlations between the mimicry score and the assessed personality scales did not yield any significant results, *r* < .15, *p* > .99. Moreover, when testing the influence of gender on mimicry, an independent sample *t*-test did not yield a significant effect either, *t*(194) = .81, *p* = .42.

#### Relation between automatic imitation and personality scales as well as demographics

Firstly, we correlated the reaction-time-based scores of the automatic imitation task with all assessed scales. The congruency effect correlated significantly with the Personality Distress subscale of the Interpersonal Reactivity Index (IRI; [52]), *r* = .26, *p* = .02, but not with any other scale (*rs* < .21, *ps* > .07). The facilitation effect did not correlate with any of the scales either (*rs* < .22, *ps* > .07). Likewise, all correlations between the interference effect and the assessed scales were non-significant (*rs* < .23, *ps* > .07).

Secondly, we tested for gender differences in the reaction-time-based imitation measures. *T*-tests for independent samples detected stronger congruency effects and facilitation effects for women (*M*_*congruency*_ = 50.96, *SD*_*congruency*_ = 29.34; *M*_*facilitation*_ = 28.19, *SD*_*facilitation*_ = 14.94) than for men (*M*_*congruency*_ = 35.61, *SD*_*congruency*_ = 27.63; *M*_*facilitation*_ = 17.94, *SD*_*facilitation*_ = 15.42), *ts*(194) > 3.45, *ps* < .002, *d*s > .53. Although not significant, there was a similar trend for the interference effect *t*(194) = 1.69, *p* = .094.

Thirdly, we ran the same analyses for the error-based automatic imitation scores. However, significant correlations were neither found for the congruency effect (*r* < .21, *p* > .22), nor for the facilitation effect (*r* < .16, *p* > .46), nor for the interference effect (*r* < .20, *p* > .38). Also, there was no gender difference for the congruency effect, the facilitation effect, or the interference effect, *t*s(194) < 1.45, *p*s > .14.

**Table 3:** Intercorrelations between all different imitation scores and all assessed scales and subscales.

	Mimicry	Congruency RT	Facilitation RT	Interference RT	Congruency ER	Facilitation ER	Interference ER
IRI PT	-0.114	-0.071	-0.036	-0.078	-0.084	0.007	-0.085
IRI FS	0.090	0.058	0.082	0.021	-0.050	0.043	-0.069
IRI EC	0.040	0.200**^†^	0.203**^†^	0.139	-0.029	0.055	-0.054
IRI PD	0.096	0.263***^†^	0.216**	0.222**^†^	0.127	-0.034	0.141*
IRI Total	0.059	0.174*	0.178*	0.119	-0.006	0.026	-0.018
SCS Independence	-0.009	-0.055	-0.003	-0.081	-0.202**	-0.012	-0.191**
SCS Interdependence	0.023	-0.161*	-0.075	-0.182*^†^	-0.119	0.043	-0.137
SCS Difference	-0.023	0.078	0.053	0.075	-0.057	-0.040	-0.037
ICS Individualism	-0.009	-0.080	-0.071	-0.063	-0.077	-0.081	-0.036
ICS Collectivism	-0.002	-0.026	0.023	-0.058	-0.107	0.138	-0.170*
ICS Difference	-0.007	-0.049	-0.073	-0.015	0.006	-0.156*	0.081
Need to Belong	0.000	0.136	0.089	0.133	0.077	0.012	0.069
AQ SS	0.031	-0.094	-0.057	-0.096	-0.121	0.007	-0.121
AQ AS	0.050	-0.067	-0.041	-0.068	-0.056	0.104	-0.104
AQ C	-0.043	-0.051	-0.071	-0.020	-0.200**	-0.128	-0.134
AQ I	0.040	0.062	0.064	0.042	-0.014	0.095	-0.059
AQ AD	0.003	0.147*	0.028	0.198**^†^	0.055	0.079	0.016
AQ Total	-0.037	-0.067	-0.078	-0.038	-0.011	-0.090	0.032
Social Engagement	0.053	0.003	-0.020	0.021	-0.064	0.079	-0.100
Objective SES	-0.030	0.115	-0.044	0.208**^†^	0.067	-0.045	0.086
Subjective SES	0.120	0.091	0.099	0.058	0.057	-0.069	0.089
Close Friends	0.097	-0.033	-0.129	0.054	-0.038	-0.087	0.004
Facebook Friends	-0.035	0.021	0.028	0.009	0.140	0.137	0.072
LS CE	0.024	-0.103	-0.126	-0.054	-0.032	0.073	-0.066
LS RO	0.142*	-0.126	-0.165*	-0.057	-0.135	-0.078	-0.094
LS AC	-0.070	-0.076	-0.060	-0.067	0.007	-0.033	0.022
LS AE	-0.011	-0.083	-0.076	-0.064	-0.083	-0.057	-0.054
Promotion Focus	-0.073	-0.036	-0.025	-0.035	0.014	0.071	-0.020
Prevention Focus	-0.073	0.002	0.042	-0.031	0.055	0.069	0.020
RF Difference	0.005	-0.037	-0.068	-0.002	-0.044	-0.004	-0.041

* p < .05

** p < .01

*** p < .001 for uncorrected multiple comparisons

^†^ = *p* < .10 for corrected multiple comparisons

*Note*. RT = Reaction Time; ER = Error Rate; PT = Perspective Taking; FS = Fantasy Scale; EC = Empathic Concern; PD = Personality Distress; SCS = Self-Construal Scale; ICS = Individualism and Collectivism Scale; AQ = Autism-Spectrum Quotient; SS = Social Skill; AS = Attention Switching; C = Communication; I = Imagination; AD = Attention to Detail; SES = Socioeconomic Status; LS = Learning Style; CE = Concrete Experience; RO = Reflective Observation; AC =Abstract Conceptualization; AE = Active Experimentation; RF = Regulatory Focus

## Discussion

When assessing individuals’ tendency to imitate others, researchers have been using different tasks. While social psychologists assess mimicry, cognitive psychologists assess automatic imitation. Both forms of imitation share similarities, but also differ in important aspects. Although it is largely assumed that mimicry and automatic imitation are grounded in similar underlying processes, the assessment of mimicry and automatic imitation differs in many methodological aspects. This raises the fundamental question to which degree these two forms of imitation are actually correlated to each other. In order to shed light on this open question, we measured mimicry as well as automatic imitation and correlated these two tasks. Moreover, we tested the relation of personality scales that have previously been reported to correlate with imitation (i.e. empathy, autism-like personality traits, traits related to self- versus other-focus), scales that have not yet been reported in the literature (i.e., regulatory focus, learning style) as well as demographics (i.e., gender, socioeconomic status, social engagement, amount of friends).

In line with past research, we detected significant imitation as well as mimicry effects. However, we did not find a significant correlation between the two tasks. This was further supported by Bayesian analyses showing that mimicry and automatic imitation were indeed not correlated. In addition, none of the assessed personality scales correlated with mimicry or automatic imitation in a meaningful way. When controlling for multiple comparisons, there was only one single significant relation, namely a correlation between the reaction time based congruency effect and the Personality Distress subscale of the Interpersonal Reactivity Index (IRI; [52]).

The absence of a correlation between mimicry and automatic imitation might be due to different reasons. First, it could be caused by differences in the methodological setup of the two paradigms. While automatic imitation is measured trial-by-trial, is based on reaction times or error rates, elicits awareness in participants, and needs explicit cognitive control, mimicry is based on behavioral observations, is rather subjectively detected, remains unaware for participants, and does not need explicit cognitive control. Although mimicry and automatic imitation did not correlate with each other, we do not argue that these two constructs are not related to each other at all. Rather, we suggest that both paradigms are part of the same construct (i.e., imitation), but measure different aspects of it. That is, while mimicry measures imitation in a social context, automatic imitation measures imitation in a laboratory context instead (for a similar distinction see [28]).

Second, another reason for the missing correlation could be found in the reliability of the two tasks. While the automatic imitation task produced good reliability, the mimicry task was not reliable at all. It is important to note that in line with past research on the mimicry task (e.g., [6,19,27]), the inter-judge reliability of coded actions was high in our study. However, as far as we know, we measured for the first time the actual reliability of the mimicry task—that is the internal consistency of mimicry performance between odd and even minutes—and detected very low reliability. Psychometric theory has shown that unreliable tasks are less likely to correlate with other tasks (cf., [93,94]). Thus, it might well be that mimicry and automatic imitation are strongly linked to each other, but that the low reliability of the mimicry task reduces the likelihood to detect such a relation. Despite the low reliability, it is important to note, however, that a reduced reliability does not necessarily indicate that the task is not replicable (e.g., [95]). Indeed, mimicry tasks have been replicated numerous times by different researchers in different labs (for an overview see [30]).

Since past research has not reported internal consistency of mimicry performance, we do not know how reliable mimicry tasks are in general. But, if mimicry tasks are indeed rather unreliable, one should interpret past correlations of this task with caution. Future research may, thus, consider other tasks that assess mimicry with multiple trials in order to achieve higher internal consistency. For example, one could use tasks that measure mimicry of facial expression. It has been found that such tasks have good sensitivity to ASD related behaviors [96], and are sensitive to social variables [97].

The second finding that most of the assessed personality scales did not correlate with mimicry and automatic imitation in a meaningful way may be surprising. There might be different reasons for such a finding. First, given that unreliable tasks are less likely to correlate with other constructs (cf., [93,94]), it should be not surprising that mimicry did not correlate with any of the assessed personality scales. Moreover, since some of the assessed scales were not very reliable either, it is also not surprising that we did not find more significant results. Second, it might be that controlling for multiple comparisons reduced statistical power and as such masked some potential effects. Indeed, when not controlling for multiple comparisons, some correlations were significant. It is important to note, however, that all correlation coefficients were very low (i.e., *r*s < .27). Since irrelevant effects can become significant with large samples (e.g., [98,99]), one should be careful not to overinterpret such low correlations. Third, it might be that the assessed scales actually do not correlate with imitation. This interpretation is in line with other recent studies that did not find significant relations between imitation and different personality scales either. For instance, Butler and Ramsey [66] did not find a relation between automatic imitation and extraversion, agreeableness, disorders of social cognition (i.e., autistic-like and schizotypal traits), narcissism, and empathy. A crucial difference between studies that found a relation with personality scales and studies that did not is the sample size. While previous studies used rather low numbers of participants, we, but also Butler and Ramsey [66], aimed for high power. As underpowered studies in combination with publication bias increase the likelihood of false positives (e.g., [99]), one should regard previous findings suggesting a relation between personality traits and imitation with caution.

Moreover, the finding that the automatic imitation task does not correlate with mimicry and most social related personality traits raises the question to which degree automatic imitation actually taps into social processes. While previous research suggests that automatic imitation relates to social factors such as eye contact [100], social attitudes [101], human-like actions (e.g., [102,103]), or a pro-social focus [57–59,101,104,105], there are also a few studies that do not support this view. For instance, some studies did not find increased automatic imitation in human as compared to non-human agents (e.g., [54,106,107]). Moreover, Butler and Ramesey [66] did not find a relationship between social components of personality traits and automatic imitation. Likewise, some researchers did not find reduced automatic imitation in individuals with autism [62–64]. Finally, Farmer et al. [108] did not find an influence of social status and power on automatic imitation. Thus, in light of these contradictory findings, more research is needed in order to draw specific conclusions about the degree to which social factors facilitate automatic imitation.

Despite the lack of correlations, there are some other findings that merit further discussion. First, we found a significant correlation between the reaction-time-based congruency effect and the Personality Distress subscale of the Interpersonal Reactivity Index (IRI; [52]). While this finding goes in line with other research suggesting a positive relationship between automatic imitation and empathy [47–49], our effect should be interpreted with caution because this relation was only significant between one subscale of the IRI and one automatic imitation score. Second, when not controlling for multiple comparisons, some correlations between automatic imitation and the assessed personality scales would actually have correlated significantly. Although due to our large sample size [98,99] the correlations that were significant at the uncorrected level should be regarded with caution, future research may follow up these correlations in order to test to which degree they are nonetheless reliable. Third, we found a significant gender difference in automatic imitation with female participants showing stronger automatic imitation effects than male participants. While this finding is in line with previous studies (e.g., [66]), it raises the question of why this is the case. It might well be that differences in personality traits account for this difference. However, personality traits including empathy, autism-spectrum quotient, and traits related to self- versus other-focus cannot explain the sex-difference, as we did not find a relation between these traits and the automatic imitation task. Thus, future research may test other personality traits that are more prone to sex-differences.

### Limitations

First, it is possible that our null results could be explained by the quality of the assessed scales. Indeed, some subscales had rather low reliability. However, most scales that have been previously found to correlate with imitation (i.e., perspective taking, self-construal, autism total scale) had an overall good reliability. Moreover, all measures that were used had been validated and are well established in the literature. Nevertheless, future research should use additional scales and scales with more reliable long-formats.

Second, one could argue that automatic imitation in the current study was confounded with spatial compatibility. To control for left-right spatial compatibility, we used a well-known method in which the stimulus hand is presented orthogonal to the response hand (e.g., [70,71,72]). Nevertheless, a potential concern with this method is that it fails to control for orthogonal spatial compatibility [109]. As a result, it is possible that orthogonal spatial processes introduced noise to the data that in turn masked potential correlations. Given that automatic imitation can still be observed even when spatial processes cannot contribute [28], future research should use tasks that control not only left-right, but also orthogonal spatial compatibility [110] in order to reduce noise in the data and as such increase statistical power.

Third, as it is common in psychological research, most of our participants were female. Although 61 male subjects is atypically high for psychological research, it may still be that the lower percentage of men, as compared to women, reduced the variability within the tasks causing null effects. In a related vein, it is important to note that all of our subjects were young psychology students. This, again, may have caused low levels of variance within the tasks, increasing the likelihood of finding null effects. Future research should, thus, aim at assessing more intermixed samples.

## Conclusion

The present study provides novel insights into the understanding of imitation. First, we found no relationship between mimicry and automatic imitation suggesting that, despite similar underlying mechanisms, the two forms of imitation are less related to each other than initially suggested. Second, we did not find meaningful relationships between any form of imitation and most personality traits including empathy, autism-like personality traits, and traits related to self- versus other-focus. Therefore, we suggest in line with other research (i.e., [66]) that the relationship between social components of personality traits and imitation is less universal than previously reported in the literature. As previous studies on the relation between personality traits and imitation tested their predictions on rather low numbers of participants, our research stresses the importance to study larger samples in order to replicate and extend previously established findings.

## References

[1] Dimberg U (1982) Facial reactions to facial expressions. Psychophysiology 19: 643–647. 7178381 10.1111/j.1469-8986.1982.tb02516.x

[2] Cappella JN, Planalp S (1981) Talk and silence sequences in informal conversations III: Interspeaker influence. Human Communication Research 7: 117–132.

[3] Giles H, Powesland PF, editors (1975) Speech style and social evaluation London: Academic Press.

[4] Webb JT (1969) Subject speed rates as a function of interviewer behavior. Language & Speech 12: 54–67. 5789296 10.1177/002383096901200105

[5] Webb JT (1972) Interview synchrony: An investigation of two speech rate measures in an automated standardized interview In: Pope B, Siegman AW, editors. Studies in dyadic communication. New York: Pergamon pp. 115–133.

[6] Chartrand TL, Bargh JA (1999) The chameleon effect: The perception-behavior link and social interaction. Journal of Personality & Social Psychology 76: 893–910. 10402679 10.1037//0022-3514.76.6.893

[7] LaFrance M (1982) Posture mirroring and rapport In: Davis M, editor. Interaction rhythms: Periodicity in communicative behavior. New York: Human Sciences Press pp. 279–298.

[8] Bernieri FJ (1988) Coordinated movement and rapport in teacher-student interactions. Journal of Nonverbal Behavior 12: 120–138.

[9] Hansen J, Alves H, Trope Y (in press) Psychological Distance Reduces Literal Imitation: Evidence From an Imitation-Learning Paradigm. Journal of Experimental Psychology: Human Perception and Performance. 10.1037/xhp0000150 26414166

[10] Brass M, Bekkering H, Wohlschläger A, Prinz W (2000) Compatibility between observed and executed finger movements: comparing symbolic, spatial, and imitative cues. Brain and Cognition 44: 124–143. doi: 10.1006/brcg.2000.1225 11041986 10.1006/brcg.2000.1225

[11] Genschow O, Florack A (2014) Attention on the Source of Influence Reverses the Impact of Cross-Contextual Imitation. Journal of Experimental Psychology: Human Perception and Performance 40: 904–907. doi: 10.1037/a0035430 24446718 10.1037/a0035430

[12] Genschow O, Florack A, Wänke M (2013) The power of movement: Evidence for context-independent movement imitation. Journal of Experimental Psychology: General 142: 763–773. 22946897 10.1037/a0029795

[13] Genschow O, Schindler S (2016) The influence of group membership on cross-contextual imitation. Psychonomic Bulletin & Review 23: 1257–1265. 26631183 10.3758/s13423-015-0983-4

[14] Hofree G, Urgen BA, Winkielman P, Saygin AP (2015) Observation and imitation of actions performed by humans, androids, and robots: an EMG study. Frontiers in human neuroscience 9. 10.3389/fnhum.2015.00364 PMC4473002 26150782

[15] Kavanagh LC, Winkielman P (2016) The Functionality of Spontaneous Mimicry and Its Influences on Affiliation: An Implicit Socialization Account. Frontiers in Psychology 7: 458 doi: 10.3389/fpsyg.2016.00458 27064398 10.3389/fpsyg.2016.00458 PMC4814497

[16] van Baaren R, Janssen L, Chartrand TL, Dijksterhuis A (2009) Where is the love? The social aspects of mimicry. Philosophical Transactions of the Royal Society B: Biological Sciences 364: 2381–2389. 10.1098/rstb.2009.0057 PMC2865082 19620109

[17] van Baaren R, Holland RW, Steenaert B, Van Knippenberg A (2003) Mimicry for money: Behavioral consequences of imitation. Journal of Experimental Social Psychology 39: 393–398.

[18] Lakin JL, Chartrand TL (2003) Using nonconscious behavioral mimicry to create affiliation and rapport. Psychological Science 14: 334–339. doi: 10.1111/1467-9280.14481 12807406 10.1111/1467-9280.14481

[19] Lakin JL, Chartrand TL, Arkin RM (2008) I am too just like you - Nonconscious mimicry as an automatic behavioral response to social exclusion. Psychological Science 19: 816–822. doi: 10.1111/j.1467-9280.2008.02162.x 18816290 10.1111/j.1467-9280.2008.02162.x

[20] Ml Stel, Blascovich J, McCall C, Mastop J, Van Baaren RB, Vonk R (2010) Mimicking disliked others: Effects of a priori liking on the mimicry-liking link. European Journal of Social Psychology 40: 867–880.

[21] Stel M, van Baaren RB, Blascovich J, van Dijk E, McCall C, Pollmann MM, et al (2010) Effects of a priori liking on the elicitation of mimicry. Experimental Psychology 57: 412–418. doi: 10.1027/1618-3169/a000050 20178935 10.1027/1618-3169/a000050

[22] van Baaren RB, Horgan TG, Chartrand TL, Dijkmans M (2004) The forest, the trees, and the chameleon: context dependence and mimicry. Journal of Personality and Social Psychology 86: 453–459. doi: 10.1037/0022-3514.86.3.453 15008648 10.1037/0022-3514.86.3.453

[23] Genschow O, Brass M (2015) The predictive chameleon: Evidence for anticipated action. Journal of Experimental Psychology: Human Perception and Performance 41: 265–268. doi: 10.1037/xhp0000035 25665086 10.1037/xhp0000035

[24] Gueguen N, Martin A, Vion M (2009) The effects of incidental similarity between two individuals on mimicry behavior. PSYCHOLOGIE FRANCAISE 54: 337–353.

[25] Hall NR, Millings A, Bouças SB (2012) Adult attachment orientation and implicit behavioral mimicry. Journal of Nonverbal Behavior 36: 235–247.

[26] van Baaren RB, Fockenberg DA, Holland RW, Janssen L, van Knippenberg A (2006) The moody chameleon: The effect of mood on non-conscious mimicry. Social Cognition 24: 426–437.

[27] Yabar Y, Johnston L, Miles L, Peace V (2006) Implicit behavioral mimicry: Investigating the impact of group membership. Journal of Nonverbal Behavior 30: 97–113.

[28] Heyes C (2011) Automatic imitation. Psychological Bulletin 137: 463–483. doi: 10.1037/a0022288 21280938 10.1037/a0022288

[29] Brass M, Bekkering H, Prinz W (2001) Movement observation affects movement execution in a simple response task. Acta Psychologica 106: 3–22. 11256338 10.1016/s0001-6918(00)00024-x

[30] Chartrand TL, Dalton AN (2009) Mimicry: Its ubiquity, importance, and functionality In: Morales E, Gollwitzer PM, Bargh JA, editors. The psychology of action: Vol 2 Mechanisms of human action: Oxford University Press pp. 893–910.

[31] van Leeuwen ML, van Baaren RB, Martin D, Dijksterhuis A, Bekkering H (2009) Executive functioning and imitation: Increasing working memory load facilitates behavioural imitation. Neuropsychologia 47: 3265–3270. doi: 10.1016/j.neuropsychologia.2009.06.005 19538976 10.1016/j.neuropsychologia.2009.06.005

[32] Greenwald AG (1970) Sensory feedback mechanisms in performance control: With special reference to the ideo-motor mechanism. Psychological Review 77: 73–99. 5454129 10.1037/h0028689

[33] Jeannerod M, Arbib MA, Rizzolatti G, Sakata H (1995) Grasping objects: the cortical mechanisms of visuomotor transformatio. Trends Neurosci 18: 314–320. 7571012

[34] Prinz W (1990) A common coding approach to perception and action In: Neumann O, Prinz W, editors. Relationships between perception and action. Berlin: Springer-Verlag pp. 167–201.

[35] Prinz W (1997) Perception and action planning. European journal of cognitive psychology 9: 129–154.

[36] Chartrand TL, Maddux WW, Lakin JL (2005) Beyond the perception-behavior link: The ubiquitous utility and motivational moderators of nonconscious mimicry In: Hassin R, Uleman J, Bargh JA, editors. Unintended thoughts 2: The new unconscious. New York: Oxford University Press pp. 334–361.

[37] Dijksterhuis A, Bargh JA (2001) The perception-behavior expressway: Automatic effects of social perception on social behavior. Advances in Experimental Social Psychology 33: 1–40.

[38] Dijksterhuis A, Chartrand TL, Aarts H (2005) The relation between perception and motivation In: Bargh JA, editor. Handbook of automaticity. Philadelphia: Psychology Press pp. 207–222.

[39] Craighero L, Bello A, Fadiga L, Rizzolatti G (2002) Hand action preparation influences the responses to hand pictures. Neuropsychologia 40: 492–502. 11749979 10.1016/s0028-3932(01)00134-8

[40] Kilner J, Paulignan Y, Blakemore S (2003) An interference effect of observed biological movement on action. Current Biology 13: 522–525. 12646137 10.1016/s0960-9822(03)00165-9

[41] Gazzola V, Keysers C (2009) The observation and execution of actions share motor and somatosensory voxels in all tested subjects: single-subject analyses of unsmoothed fMRI data. Cerebral Cortex 19: 1239–1255. doi: 10.1093/cercor/bhn181 19020203 10.1093/cercor/bhn181 PMC2677653

[42] Keysers C, Gazzola V (2010) Social neuroscience: mirror neurons recorded in humans. Current Biology 20: 353–354. doi: 10.1016/j.cub.2009.12.050 21749952 10.1016/j.cub.2010.03.013

[43] Catmur C, Walsh V, Heyes C (2007) Sensorimotor learning configures the human mirror system. Current Biology 17: 1527–1531. doi: 10.1016/j.cub.2007.08.006 17716898 10.1016/j.cub.2007.08.006

[44] Fadiga L, Fogassi L, Pavesi G, Rizzolatti G (1995) Motor facilitation during action observation: a magnetic stimulation study. Journal of Neurophysiology 73: 2608–2611. 7666169 10.1152/jn.1995.73.6.2608

[45] Di Pellegrino G, Fadiga L, Fogassi L, Gallese V, Rizzolatti G (1992) Understanding motor events: a neurophysiological study. Experimental Brain Research 91: 176–180. 1301372 10.1007/BF00230027

[46] Mukamel R, Ekstrom AD, Kaplan J, Iacoboni M, Fried I (2010) Single-neuron responses in humans during execution and observation of actions. Current biology 20: 750–756. doi: 10.1016/j.cub.2010.02.045 20381353 10.1016/j.cub.2010.02.045 PMC2904852

[47] Müller BC, Leeuwen ML, Baaren RB, Bekkering H, Dijksterhuis A (2013) Empathy is a beautiful thing: Empathy predicts imitation only for attractive others. Scandinavian journal of psychology 54: 401–406. doi: 10.1111/sjop.12060 23786165 10.1111/sjop.12060

[48] Sonnby-Borgström M, Jönsson P, Svensson O (2003) Emotional empathy as related to mimicry reactions at different levels of information processing. Journal of Nonverbal behavior 27: 3–23.

[49] Sonnby–Borgström M (2002) Automatic mimicry reactions as related to differences in emotional empathy. Scandinavian journal of psychology 43: 433–443. 12500783 10.1111/1467-9450.00312

[50] Jackson PL, Meltzoff AN, Decety J (2006) Neural circuits involved in imitation and perspective-taking. Neuroimage 31: 429–439. doi: 10.1016/j.neuroimage.2005.11.026 16406257 10.1016/j.neuroimage.2005.11.026 PMC1475952

[51] Lamm C, Batson CD, Decety J (2007) The neural substrate of human empathy: Effects of perspective-taking and cognitive appraisal. Journal of Cognitive Neuroscience 19: 42–58. doi: 10.1162/jocn.2007.19.1.42 17214562 10.1162/jocn.2007.19.1.42

[52] Davis MH (1980) A multidimensional approach to individual differences in empathy. JSAS Catalog of Selected Documents in Psychology 10: 85.

[53] Horton WS (2014) Individual differences in perspective taking and field-independence mediate structural persistence in dialog. Acta psychologica 150: 41–48. doi: 10.1016/j.actpsy.2014.04.006 24816270 10.1016/j.actpsy.2014.04.006

[54] Cracco E, De Coster L, Andres M, Brass M (2015) Motor simulation beyond the dyad: Automatic imitation of multiple actors. Journal of Experimental Psychology: Human Perception and Performance 41: 1488–1501, Empathy results retrieved from osf.io/eas1484m. doi: 10.1037/a0039737 26389616 10.1037/a0039737

[55] Cracco E, Brass M (2017, May 29) Motor simulation beyond the dyad: Automatic imitation of multiple actors. Retrieved from osfio/eas4m 10.1037/a0039737 26389616

[56] Brewer MB (1991) The social self: On being the same and different at the same time. Personality and social psychology bulletin 17: 475–482.

[57] van Baaren RB, Maddux WW, Chartrand TL, De Bouter C, van Knippenberg A (2003) It takes two to mimic: behavioral consequences of self-construals. Journal of personality and social psychology 84: 1093–1102. 12757151 10.1037/0022-3514.84.5.1093

[58] Hogeveen J, Obhi SS (2011) Altogether now: activating interdependent self-construal induces hypermotor resonance. Cognitive neuroscience 2: 74–82. doi: 10.1080/17588928.2010.533164 24168476 10.1080/17588928.2010.533164

[59] Spengler S, Brass M, Kühn S, Schütz-Bosbach S (2010) Minimizing motor mimicry by myself: self-focus enhances online action-control mechanisms during motor contagion. Consciousness and cognition 19: 98–106. doi: 10.1016/j.concog.2009.12.014 20116291 10.1016/j.concog.2009.12.014

[60] Cook J, Swapp D, Pan X, Bianchi-Berthouze N, Blakemore S-J (2014) Atypical interference effect of action observation in autism spectrum conditions. Psychological medicine 44: 731–740. doi: 10.1017/S0033291713001335 23759288 10.1017/S0033291713001335 PMC3898726

[61] Williams JH, Whiten A, Singh T (2004) A systematic review of action imitation in autistic spectrum disorder. Journal of autism and developmental disorders 34: 285–299. 15264497 10.1023/b:jadd.0000029551.56735.3a

[62] Bird G, Leighton J, Press C, Heyes C (2007) Intact automatic imitation of human and robot actions in autism spectrum disorders. Proceedings of the Royal Society of London B: Biological Sciences 274: 3027–3031. 10.1098/rspb.2007.1019 PMC2291158 17911053

[63] Gowen E, Stanley J, Miall R (2008) Movement interference in autism-spectrum disorder. Neuropsychologia 46: 1060–1068. doi: 10.1016/j.neuropsychologia.2007.11.004 18096192 10.1016/j.neuropsychologia.2007.11.004 PMC6010145

[64] Press C, Richardson D, Bird G (2010) Intact imitation of emotional facial actions in autism spectrum conditions. Neuropsychologia 48: 3291–3297. doi: 10.1016/j.neuropsychologia.2010.07.012 20638398 10.1016/j.neuropsychologia.2010.07.012 PMC3221037

[65] Spengler S, Bird G, Brass M (2010) Hyperimitation of actions is related to reduced understanding of others' minds in autism spectrum conditions. Biological Psychiatry 68: 1148–1155. doi: 10.1016/j.biopsych.2010.09.017 21130224 10.1016/j.biopsych.2010.09.017

[66] Butler EE, Ward R, Ramsey R (2015) Investigating the Relationship between Stable Personality Characteristics and Automatic Imitation. PloS one 10: e0129651 doi: 10.1371/journal.pone.0129651 26079137 10.1371/journal.pone.0129651 PMC4469457

[67] Osborne JW, Overbay A (2004) The power of outliers (and why researchers should always check for them). Practical assessment, research & evaluation 9: 1–12.

[68] Frohlich F, Vogl C (2010) 365 dierenverhaaltjes - Konijnen. Noordwijkerhout, Netherlands: Rebo Productions.

[69] Stevens M, Lammertyn J, Verbruggen F, Vandierendonck A (2006) Tscope: AC library for programming cognitive experiments on the MS Windows platform. Behavior Research Methods 38: 280–286. 16956104 10.3758/bf03192779

[70] Cook J, Bird G (2011) Social attitudes differentially modulate imitation in adolescents and adults. Experimental Brain Research 211: 601–612. doi: 10.1007/s00221-011-2584-4 21336831 10.1007/s00221-011-2584-4 PMC3102210

[71] Cook JL, Bird G (2012) Atypical social modulation of imitation in autism spectrum conditions. Journal of autism and developmental disorders 42: 1045–1051. doi: 10.1007/s10803-011-1341-7 21833823 10.1007/s10803-011-1341-7 PMC3360861

[72] Jiménez L, Recio S, Méndez A, Lorda MJ, Permuy B, Méndez C (2012) Automatic imitation and spatial compatibility in a key-pressing task. Acta psychologica 141: 96–103. doi: 10.1016/j.actpsy.2012.07.007 22864312 10.1016/j.actpsy.2012.07.007

[73] De Corte K, Buysse A, Verhofstadt LL, Roeyers H, Ponnet K, Davis MH (2007) Measuring empathic tendencies: Reliability and validity of the Dutch version of the Interpersonal Reactivity Index. Psychologica Belgica 47: 235–260.

[74] Singelis TM (1994) The measurement of independent and interdependent self-construals. Personality and social psychology bulletin 20: 580–591.

[75] Triandis HC, Gelfand MJ (1998) Converging measurement of horizontal and vertical individualism and collectivism. Journal of personality and social psychology 74: 118–128.

[76] Pickett CL, Gardner WL, Knowles M (2004) Getting a cue: The need to belong and enhanced sensitivity to social cues. Personality and Social Psychology Bulletin 30: 1095–1107. doi: 10.1177/0146167203262085 15359014 10.1177/0146167203262085

[77] Leary MR, Kelly KM, Cottrell CA, Schreindorfer LS (2013) Construct validity of the need to belong scale: Mapping the nomological network. Journal of personality assessment 95: 610–624. doi: 10.1080/00223891.2013.819511 23905716 10.1080/00223891.2013.819511

[78] Hoekstra RA, Bartels M, Cath DC, Boomsma DI (2008) Factor structure, reliability and criterion validity of the Autism-Spectrum Quotient (AQ): a study in Dutch population and patient groups. Journal of autism and developmental disorders 38: 1555–1566. doi: 10.1007/s10803-008-0538-x 18302013 10.1007/s10803-008-0538-x PMC2516538

[79] Baron-Cohen S, Wheelwright S, Skinner R, Martin J, Clubley E (2001) The autism-spectrum quotient (AQ): Evidence from asperger syndrome/high-functioning autism, malesand females, scientists and mathematicians. Journal of autism and developmental disorders 31: 5–17. 11439754 10.1023/a:1005653411471

[80] Woodbury-Smith MR, Robinson J, Wheelwright S, Baron-Cohen S (2005) Screening adults for Asperger syndrome using the AQ: A preliminary study of its diagnostic validity in clinical practice. Journal of autism and developmental disorders 35: 331–335. 16119474 10.1007/s10803-005-3300-7

[81] Griskevicius V, Tybur JM, Delton AW, Robertson TE (2011) The influence of mortality and socioeconomic status on risk and delayed rewards: a life history theory approach. Journal of personality and social psychology 100: 1015–1026. doi: 10.1037/a0022403 21299312 10.1037/a0022403 PMC3298774

[82] Kolb DA (2014) Experiential learning: Experience as the source of learning and development In: Sternberg RJ, Zhang LF, editors. Perspectives on cognitive, learning, and thinking styles. NJ: Lawrence Erlbaum.

[83] Higgins ET (1997) Beyond pleasure and pain. American psychologist 52: 1280–1300. 9414606 10.1037//0003-066x.52.12.1280

[84] Pfattheicher S, Sassenrath C (2014) A regulatory focus perspective on eating behavior: how prevention and promotion focus relates to emotional, external, and restrained eating. Frontiers in psychology 5. 10.3389/fpsyg.2014.01314 PMC4238324 25477840

[85] Lockwood P, Jordan CH, Kunda Z (2002) Motivation by positive or negative role models: regulatory focus determines who will best inspire us. Journal of Personality and Social Psychology 83: 854–864. 12374440

[86] Pennington GL, Roese NJ (2003) Regulatory focus and temporal distance. Journal of Experimental Social Psychology 39: 563–576.

[87] Shah J, Higgins T, Friedman RS (1998) Performance incentives and means: how regulatory focus influences goal attainment. Journal of Personality and Social Psychology 74: 285–293. 9491583 10.1037//0022-3514.74.2.285

[88] Uskul AK, Sherman DK, Fitzgibbon J (2009) The cultural congruency effect: Culture, regulatory focus, and the effectiveness of gain-vs. loss-framed health messages. Journal of Experimental Social Psychology 45: 535–541.

[89] Love J, Selker R, Marsman M, Jamil T, Dropmann D, Verhagen A, et al (2015) JASP (Version 0.7)[Computer software]. Amsterdam, The Netherlands: JASP Project Retrieved from https://jasp-stats.org.

[90] Dienes Z (2011) Bayesian versus orthodox statistics: Which side are you on? Perspectives on Psychological Science 6: 274–290. doi: 10.1177/1745691611406920 26168518 10.1177/1745691611406920

[91] Jeffreys H (1961) Theory of probability Clarendon Press, Oxford.

[92] Benjamini Y, Hochberg Y (1995) Controlling the false discovery rate: a practical and powerful approach to multiple testing. Journal of the royal statistical society Series B (Methodological): 289–300.

[93] Crocker L, Algina J (1986) Introduction to classical and modern test theory Fort Worth, TX: Holt, Rinehart, & Winston.

[94] Cronbach LJ (1990) Essentials of psychological testing (5th ed.). New York: Harper & Row.

[95] De Schryver M, Hughes S, Rosseel Y, De Houwer J (2015) Unreliable Yet Still Replicable: A Comment on LeBel and Paunonen (2011). Frontiers in psychology 6. 10.3389/fpsyg.2015.02039 PMC4710742 26793150

[96] McIntosh DN (2006) Spontaneous facial mimicry, liking and emotional contagion. Polish Psychological Bulletin 37: 31–42.

[97] Hess U, Fischer A (2013) Emotional mimicry as social regulation. Personality and Social Psychology Review 17: 142–157. doi: 10.1177/1088868312472607 23348982 10.1177/1088868312472607

[98] Cohen J (1969) Statistical power analysis for the behavioural sciences New York: Academic Press.

[99] Lakens D, Evers ER (2014) Sailing from the seas of chaos into the corridor of stability practical recommendations to increase the informational value of studies. Perspectives on Psychological Science 9: 278–292. doi: 10.1177/1745691614528520 26173264 10.1177/1745691614528520

[100] Wang Y, Newport R, Hamilton AFdC (2011) Eye contact enhances mimicry of intransitive hand movements. Biology Letters: 7–10. doi: 10.1098/rsbl.2010.0279 20427328 10.1098/rsbl.2010.0279 PMC3030861

[101] Leighton J, Bird G, Orsini C, Heyes C (2010) Social attitudes modulate automatic imitation. Journal of Experimental Social Psychology 46: 905–910.

[102] Liepelt R, Brass M (2010) Top-down modulation of motor priming by belief about animacy. Experimental Psychology 57: 221–227. doi: 10.1027/1618-3169/a000028 20178950 10.1027/1618-3169/a000028

[103] Longo MR, Bertenthal BI (2009) Attention modulates the specificity of automatic imitation to human actors. Experimental Brain Research 192: 739–744. doi: 10.1007/s00221-008-1649-5 19034438 10.1007/s00221-008-1649-5

[104] Leighton J, Bird G, Charman T, Heyes C (2008) Weak imitative performance is not due to a functional ‘mirroring’deficit in adults with Autism Spectrum Disorders. Neuropsychologia 46: 1041–1049. doi: 10.1016/j.neuropsychologia.2007.11.013 18177677 10.1016/j.neuropsychologia.2007.11.013

[105] Wang Y, Hamilton A (2013) Understanding the role of the ‘self’in the social priming of mimicry. PloS one 8: e60249 doi: 10.1371/journal.pone.0060249 23565208 10.1371/journal.pone.0060249 PMC3614954

[106] Jansson E, Wilson AD, Williams JH, Mon-Williams M (2007) Methodological problems undermine tests of the ideo-motor conjecture. Experimental Brain Research 182: 549–558. doi: 10.1007/s00221-007-1013-1 17593359 10.1007/s00221-007-1013-1

[107] Klapper A, Ramsey R, Wigboldus D, Cross ES (2014) The control of automatic imitation based on Bottom–Up and Top–Down cues to animacy: Insights from brain and behavior. Journal of cognitive neuroscience 26: 2503–2513. doi: 10.1162/jocn_a_00651 24742157 10.1162/jocn_a_00651

[108] Farmer H, Carr EW, Svartdal M, Winkielman P, Hamilton AFdC (2016) Status and power do not modulate automatic imitation of intransitive hand movements. PloS one 11: e0151835 doi: 10.1371/journal.pone.0151835 27096167 10.1371/journal.pone.0151835 PMC4838218

[109] Weeks DJ, Proctor RW (1990) Salient-features coding in the translation between orthogonal stimulus and response dimensions. Journal of Experimental Psychology: General 119: 355–366.

[110] Catmur C, Heyes C (2011) Time course analyses confirm independence of imitative and spatial compatibility. Journal of Experimental Psychology: Human Perception and Performance 37: 409–421. doi: 10.1037/a0019325 20731523 10.1037/a0019325

